# Spontaneous Necrotizing Fasciitis

**DOI:** 10.7759/cureus.11880

**Published:** 2020-12-03

**Authors:** Patrick J Rogers, Brent M Lewis, Mihir Odak, Joshua Bucher

**Affiliations:** 1 Emergency Medicine, Jersey Shore University Medical Center, Neptune City, USA; 2 Emergency Medicine, Community Medical Center, Toms River, USA; 3 Internal Medicine, Jersey Shore University Medical Center, Neptune City, USA; 4 Emergency Medicine, Robert Wood Johnson University Hospital, New Brunswick, USA

**Keywords:** necrotizing fascitis, soft tissue infection, surgical debridement

## Abstract

Necrotizing soft tissue infections typically begin with direct inoculation of bacteria into the subcutaneous tissues. Here, we present a case with no such exposure, but with severe necrotizing fasciitis. We present a middle-aged man presented to the emergency department for a presumed allergic reaction after having initially sought care twice at an urgent care facility. The patient had swelling, but no tenderness of his right lateral chest and flank. Subsequent imaging showed extensive fluid in the fascial planes of the right chest wall requiring surgical debridement. Necrotizing fasciitis that is not treated with surgical debridement carries a mortality rate approaching 100%. This case highlights a potential atypical presentation as well as highlights the fact that the Laboratory Risk Indicator for Necrotizing Fasciitis (LRINEC) score lacks sensitivity to rule out a necrotizing soft tissue infection, requiring surgical debridement for diagnosis.

## Introduction

Necrotizing soft tissue infections contain a large spectrum of illnesses characterized by soft tissue necrosis, systemic toxicity, and high mortality [1]. Early in the disease course, however, these infections can appear benign and their diagnosis largely involves a high degree of clinical suspicion based on history and physical examination.

The pathophysiology of necrotizing soft tissue infections begins with an event that leads to a direct invasion of subcutaneous tissue. Most commonly this involves processes associated with external trauma, introducing cutaneous and environmental bacteria into the body; however, bacteria from internal organs are described in the literature, typically involving the gallbladder [2] or appendix [3,4]. Case reports also exist suggesting the presence of gastrointestinal ulcers as potential means to communicate bacteria into the subcutaneous tissues [5].

Cases of spontaneous necrotizing soft tissue infections are deceptively reported in the literature. Aside from the rare circumstances already presented, cases are often presented as “spontaneous” while having a remote history of trauma that has been minimized or not initially revealed [6-8] or take place in patients with histories of significant immunosuppression [7]. These examples do not represent the spontaneous entrance of necrotizing bacteria into the subcutaneous tissue without any discernable entry point. This case is important and adds to the literature as it is an example of a spontaneous presentation without a history of external trauma to introduce bacteria.

## Case presentation

A previously healthy 47-year-old male with no past medical history presented to an urgent care facility four days prior and was diagnosed with a suspected right latissimus muscle tear after experiencing mild right lateral upper back and flank pain with movement of his right shoulder while working as a mechanic and performing manual labor. The patient denied any fevers, chills, trauma, rashes, skin breakdown, or wounds. He was prescribed naproxen and metaxalone for his symptoms.

On the day of presentation, the patient first returned to the urgent care facility due to intermittent headaches, nausea, and vomiting along with dyspnea and a diffuse rash covering his chest, back, groin, arms, and legs that began after taking the medications that had been prescribed four days prior to presentation. He was found to be hypotensive and tachycardic at the urgent care facility and was treated with 2.5 liters of normal saline, 0.3 mg of 1:1,000 epinephrine intramuscularly twice, 125 mg of methylprednisolone intravenously (IV), 20 mg of famotidine IV, and 50 mg of diphenhydramine IV for a presumed anaphylactic reaction and transferred to the ED with no improvement.

Upon arrival to our ED, the patient was noted to be lethargic, tachycardic, and tachypneic. Initial vital signs were a blood pressure 112/59 mmHg, pulse 132 beats per minute, respirations of 24 breaths per minute, a temperature of 97.9 degrees Fahrenheit, and oxygen saturation of 94% via nasal cannula. Pertinent positives on physical examination were scleral icterus, a diffuse erythematous macular rash with a sandpaper-like texture of the arms, trunk, groin, and right leg, with sparing of the palms and soles. There were no bullae, vesicles, drainage, or crepitus. The patient grimaced with pain when removing the shirt off his right shoulder and there was mild swelling of the right axilla and right lateral chest wall; however, there was no tenderness to palpation of the shoulder, flank, or back.

Due to lethargy and increased work of breathing the patient was intubated shortly after evaluation. The family noted that the patient worked as an automobile mechanic and had no recent travel, no trauma, exposure to wooded areas, or sick contacts and confirmed that the patient’s recent symptoms of fevers, headache, and rash began only since first going to the urgent care center four days prior. The family confirmed the patient had no history of smoking, alcohol, or substance abuse. Empiric antibiotic coverage was administered with vancomycin, piperacillin/tazobactam, clindamycin, and doxycycline, and CT scans of the head, neck, and chest/abdomen/pelvis were ordered.

**Figure 1:**
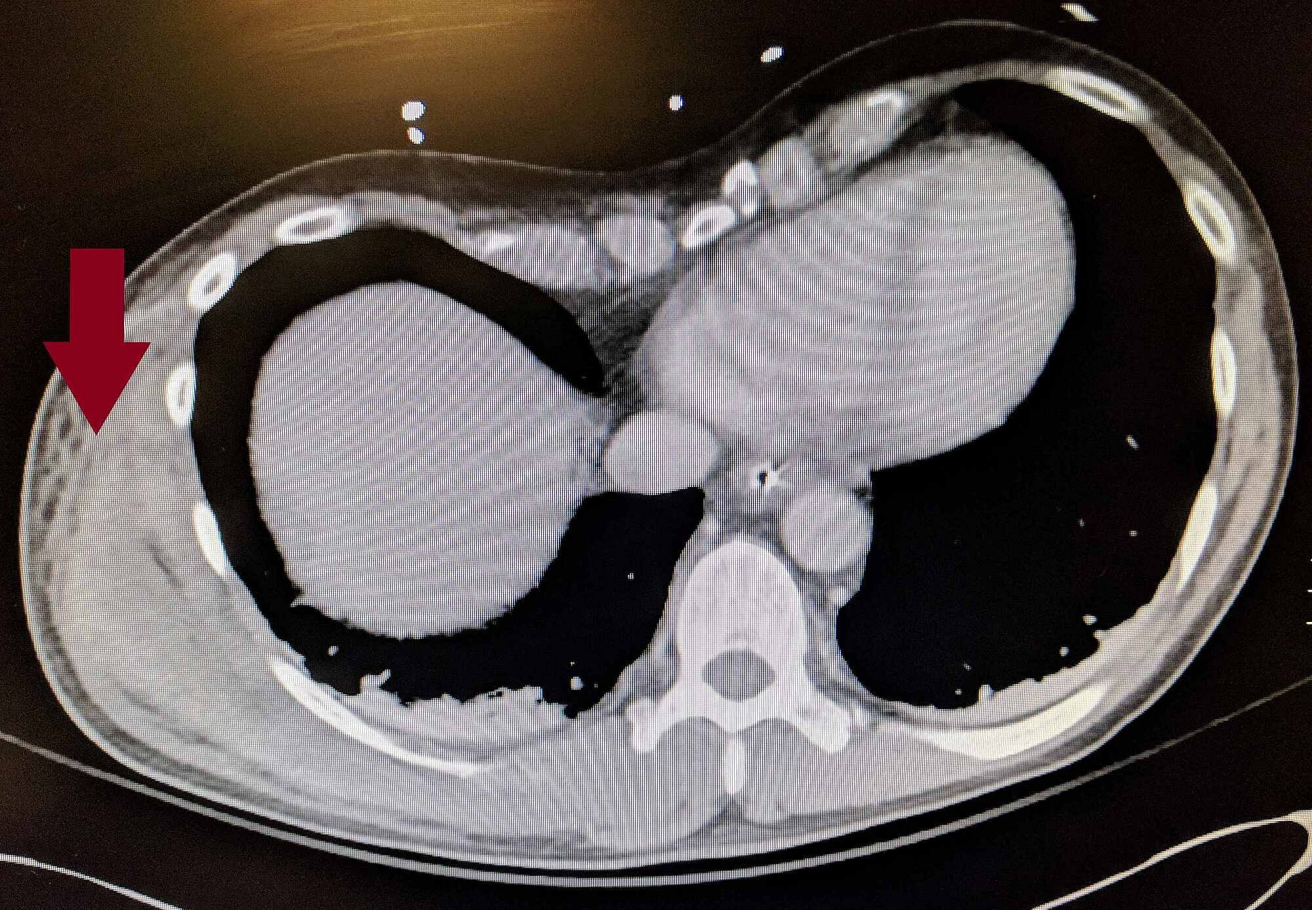
CT chest, abdomen, pelvis revealing fluid in the fascial planes in the right lateral chest wall.

**Figure 2:**
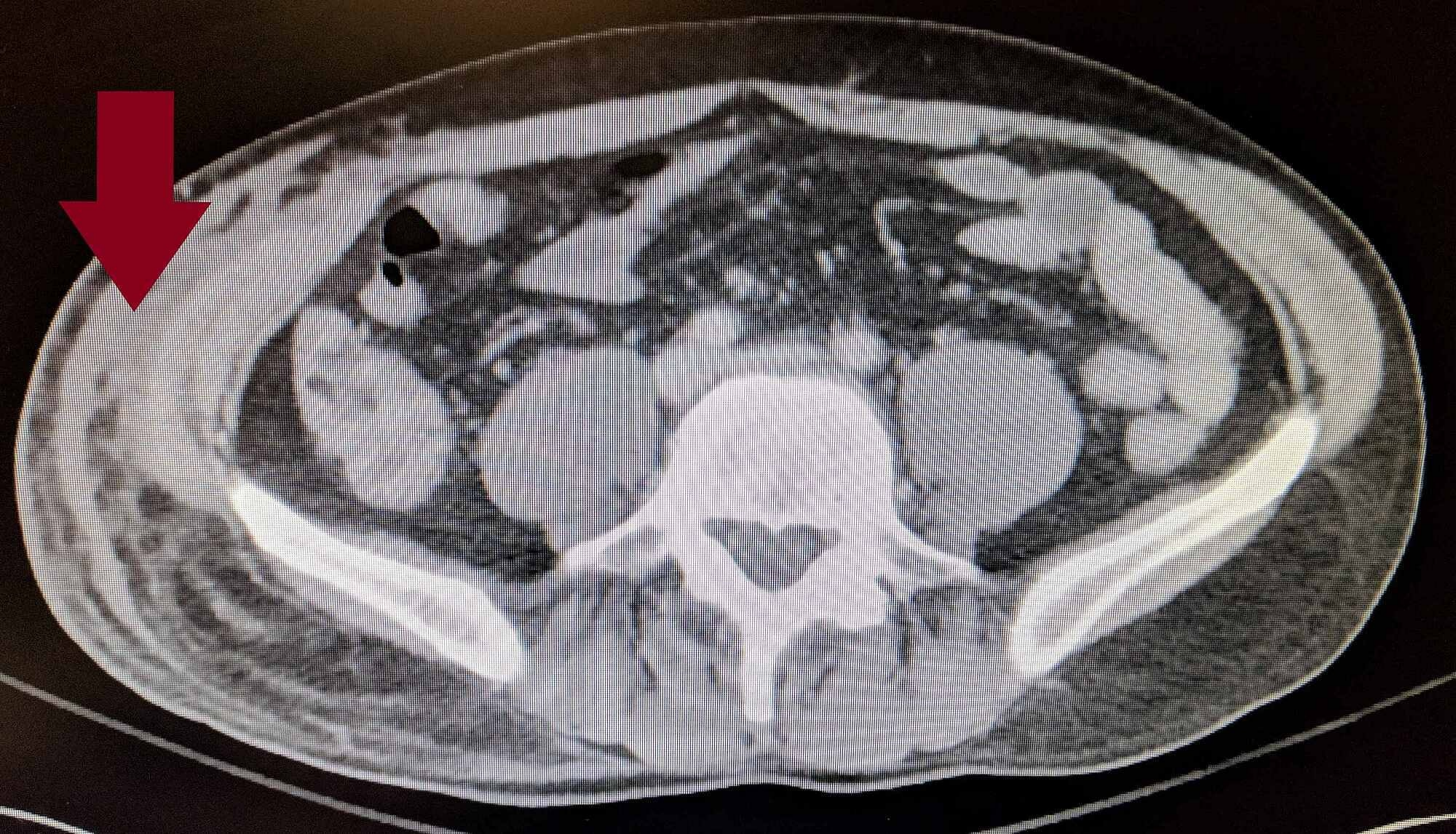
CT chest, abdomen, pelvis revealing collection of fluid in the fascial planes in the pelvic region.

**Figure 3:**
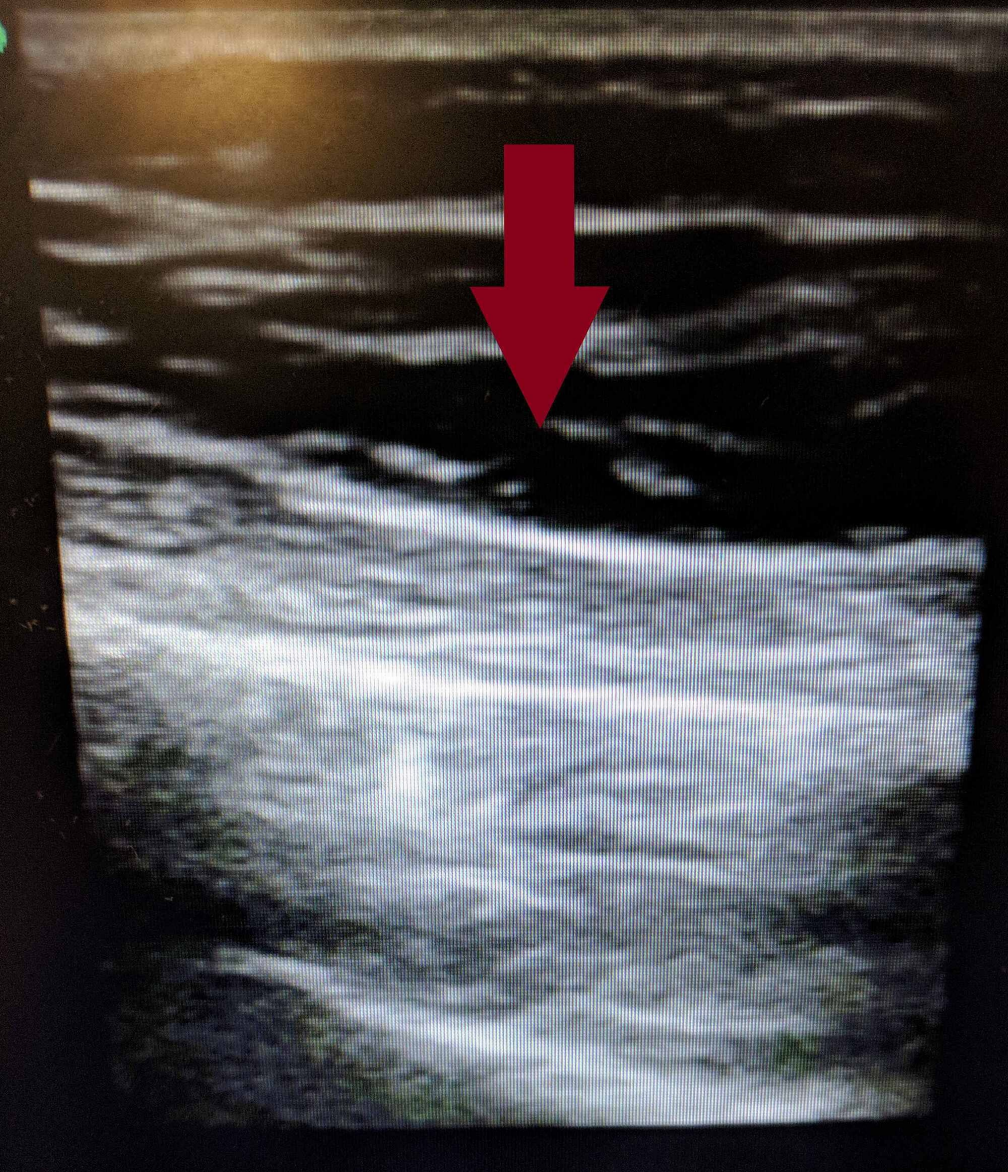
Bedside ultrasound revealing fluid in the fascial planes of the right chest wall.

After review of the CT images (Figures 1, 2), a bedside ultrasound revealed fluid in the fascial planes along the right lateral chest wall consistent with the CT scan finding of fluid and edema of the right chest wall tissues with no discrete abscess (Figure 3). Initial laboratory evaluation is shown in Table 1.

**Table 1:** Laboratory Data WBC: white blood cells; CRP: C-reactive protein; BUN: blood urea nitrogen

Laboratory Data	Value	Reference
WBC	8.6 x10^3^/uL	4.5–11.0 x10^3^/uL
Bands	26%	5–11%
Hemoglobin	14.3 mg/dL	12.0–16.0 mg/dL
Sodium	119 mmol/L	136–145 mmol/L
CRP	30.65 mg/dL	0.00–0.74 mg/dL
Chloride	82 mmol/L	96–110 mmol/L
Bicarbonate	17.2 mmol/L	24–31 mmol/L
Anion Gap	20 mmol/L	5–13 mmol/L
BUN	47 mg/dL	5–25 mg/dL
Creatinine	4.1 mg/dL	0.44–1.00 mg/dL
Calcium	7.6 mg/dL	8.5–10.5 mg/dL
Glucose	121 mg/dL	70–99 mg/dL
Lactic Acid	4.1 mmol/L	0.5–2.0 mmol/L

Given the patient’s constellation of clinical symptoms, imaging, and laboratory results, there was high clinical suspicion for necrotizing fasciitis. A surgical consult was obtained who emergently took the patient to the operating room for surgical fasciotomy and debridement. The initial surgical exploration revealed necrotic appearing latissimus dorsi as well as purulent fluid between the latissimus dorsi and the external oblique muscle. Surgical wound cultures grew Group A *Streptococcus pyogenes*. The patient subsequently had a 47-day hospitalization that involved 14 additional operative debridements but did not require amputation or bone removal. His antibiotics were changed to clindamycin and piperacillin/tazobactam and subsequently to clindamycin and penicillin VK. After cultures grew Group A *S. pyogenes*, he was continued on penicillin monotherapy. He eventually underwent extensive skin grafting to close the debrided areas and was discharged to a subacute rehab facility.

## Discussion

We present here the case of a patient without a history of external trauma who was found to have necrotizing fasciitis presumed to be of spontaneous origin. While other cases in the literature may have had a remote history of trauma, there was none in the case that was reported by the patient or the family.

*S. pyogenes* is an extremely common bacteria that contributes to a large variety of infectious processes. Necrotizing soft tissue infections are rarer, with an incidence in the developed world of approximately three cases per 100,000, though with an associated mortality approaching 50% [9-12]. Necrotizing fasciitis has an incidence of 0.3 to 15 cases per 1,000,000 [13]. Necrotizing fasciitis infections can be mono or polymicrobial and involve both aerobic and anaerobic organisms. This includes *Clostridium*, *Staphylococcus*, *Streptococcus*, *Escherichia coli*, *Fusobacterium*, *Bacteroides*, and others. Direct invasion of subcutaneous tissues is generally attributed in the literature to surgical incisions, burns, insect bites, or lesions associated with rashes such as varicella, though in varying percentages of cases the source is not identifiable [13,14].

Aside from the breakdown of cutaneous membranes, an association between nonsteroidal anti-inflammatory drugs (NSAIDs) and the development of group A Streptococcal necrotizing soft tissue infections has been suggested, possibly related to up-regulation of tumor necrosis factor-alpha and suppression of neutrophils [15]. This evidence is controversial, and several arguments exist attributing the masking of symptoms as responsible for the association with NSAID use [16,17].

The Laboratory Risk Indicator for Necrotizing Fasciitis (LRINEC) (Table 2) was established in 2004 as a clinical decision tool in evaluating soft tissue infections [18], with subsequent validation studies in 2017 [19]. In this scoring system, patients with a score of at least six should be carefully evaluated for the presence of necrotizing soft tissue infection, but it lacks sensitivity to rule out a necrotizing infection. Therefore, a lower LRINEC score should not be used to definitively rule out a necrotizing infection.

**Table 2:** Laboratory Risk Indicator for Necrotizing Fasciitis CRP: C-reactive protein; WBC: white blood cells

CRP	<15 mg/dL	0
	>=15 mg/dL	+4
WBC	<15 × 10^3^/uL	0
	15–25 × 10^3^/uL	+1
	>25 × 10^3^/uL	+2
Hemoglobin	>13.5 g/dL	0
	11–13.5 g/dL	+1
	<11 g/dL	+2
Sodium	>=135 mEq/L	0
	<135 mEq/L	+2
Creatinine	>=1.6 mg/dL	0
	>1.6 mg/dL	+2
Glucose	>=180 mg/dL	0
	>180 mg/dL	+1
Composite Risk Score	0–6	Low Risk
	6–7	Intermediate Risk
	>7	High Risk

While the initial study suggested impressive specificity, the validation study noted that all of the metrics included in the score, specifically the C-reactive protein (CRP), are often not initially obtained. An additional 2015 study suggests that adding “pain out of proportion” significantly increases the specificity, but also places increased significance on the measurement of CRP [20]. Given the labs obtained, our patient’s LRINEC score was 8, which is a high risk for necrotizing fasciitis.

Imaging may be helpful, but is not required as direct inspection via surgical exploration is the only way to rule out a necrotizing soft tissue infection. Antibiotic treatment to cover for methicillin-resistant *Staphylococcus aureus *(MRSA), Gram-negative, and anaerobic organisms is indicated; retrospective cohort studies have shown efficacy with clindamycin in treating Group A *Streptococcus* infections, however, the definitive treatment is surgical intervention [1].

## Conclusions

Necrotizing fasciitis carries a median mortality rate of 32.2%, though reported rates vary tremendously through the literature. Independent of this, however, is that necrotizing fasciitis that is not treated with surgical debridement has a mortality that approaches 100%. Specifically, because of this, necrotizing fasciitis is a cannot-miss diagnosis.

In patients with a defined point of bacteria entry, a physical examination is significant for significant pain, swelling, and erythema of the affected area. Considering the fact that this is not always the case, pain out of proportion to the exam as well as crescendo pain becomes an extremely important component of serial physical examination. While the LRINEC score can be helpful in the clinical decision-making process, the physical exam findings, including vital sign abnormalities, are the most important factors in the approach to the patient with a soft tissue infection and should function as the most significant factor in early surgical consultation for bacterial source control. Antibiotic treatment with MRSA, Gram-negative, and anaerobic coverage, while indicated, does not take the place of surgical debridement.
